# Effect of Long-Distance Running on Inter-segment Foot Kinematics and Ground Reaction Forces: A Preliminary Study

**DOI:** 10.3389/fbioe.2022.833774

**Published:** 2022-03-04

**Authors:** Jialin Li, Yang Song, Rongrong Xuan, Dong Sun, Ee-Chon Teo, István Bíró, Yaodong Gu

**Affiliations:** ^1^ The Affiliated Hospital of Medical School, Ningbo University, Ningbo, China; ^2^ Ningbo University School of Medicine, Ningbo University, Ningbo, China; ^3^ Research Academy of Human Biomechanics, Ningbo University, Ningbo, China; ^4^ Doctoral School on Safety and Security Sciences, Óbuda University, Budapest, Hungary; ^5^ Faculty of Engineering, University of Szeged, Szeged, Hungary

**Keywords:** long-distance running, foot kinematics, kinetics, multi-segment foot model, running-related injuries

## Abstract

Long-distance running has gained massive popularity in recent years, yet the intra-foot adaptations during this event remain unclear. This study aimed to examine the kinematic and ground reaction force alterations induced within the foot following a 5 and 10 km run using the Oxford Foot Model Ten marathon-experienced recreational runners participated in this study. Five-kilometer running led to more rearfoot dorsiflexion, rearfoot eversion, and rearfoot rotation while less forefoot plantarflexion during the stance phase. Increased rearfoot plantarflexion, while decreased forefoot plantarflexion, supination, adduction, and hallux plantarflexion were observed at 10 km. In addition, the forefoot space of footwear was found to play a role in hallux kinematics. Concerning GRFs, only a lesser propulsive force was presented after a 10 km run. Findings of this study showed that 5 km of running would induce excessive foot motion while 10 km of running may gradually change the foot posture and lead to reduced propulsive forces, which could potentially increase the risks of running-related injuries (RRI) due to overuse or fatigue. Nevertheless, further research is warranted, and this study could be used as a preliminary reference to evaluate and predict foot running-related injuries.

## 1 Introduction

As one of the most accessible sports to achieve better physical health and prevent diseases, running, especially long-distance running, has attracted extensive participation worldwide (Kim et al., 2018; Mei et al., 2018). It has been reported that the number of runners has doubled, and the number of marathon finishers has shown an exponential increase over the past decade (Nikolaidis et al., 2021; van Poppel et al., 2021). For instance, at least 344,000 marathon runners finished the “New York City Marathon” from 2010 to 2017, which is more than 10 times compared to ∼25,000 in 1970–1979 (Vitti et al., 2020). In China, a total of 2.8 million people participated in long-distance running races across the country in 2016, which hit a record high at the time (ChinaDaily, 2016). Unfortunately, broad participation in long-distance repetitive exercise may also bring in a higher rate of running-related injuries (RRI), particularly to the lower extremities and the foot (Van Gent et al., 2007; Hulme et al., 2017; Mei et al., 2019).

As the primary interface of the lower limb with the external environment, the foot has been previously demonstrated to be a common injury site (Kindred et al., 2011; Zhou et al., 2019; Dempster et al., 2021). In addition, some foot biomechanical changes during running may further potentially increase the risk of RRI of the lower limbs (Mei et al., 2018; Takabayashi et al., 2018; Mei et al., 2019; Matias et al., 2020). For example, increased foot pronation during running would contribute to a significantly higher medical knee contact force, consequently increasing knee injury risks (Mei et al., 2019). Different landing techniques, i.e., rearfoot strike and forefoot strike, would lead to relatively distinct foot joint rotations and bone orientations, which may further be associated with different RRI (Matias et al., 2020). Moreover, excessive rearfoot eversion angles exhibited during running would further result in excessive dorsiflexion of midfoot due to kinematic coupling. This abnormal foot motion has been proved to be a risk factor for lower limb RRI (Takabayashi et al., 2018). The above evidence clearly showed that the human foot is a particularly deformable and vulnerable structure during long-distance running. Further analysis of the foot biomechanics is therefore of great importance. At this time, numerous foot models are available for gait analysis. However, it is assumed that, based on previous studies, a multi-segment foot model is required for the in-depth foot kinematics analysis (Wolf et al., 2008; Dixon et al., 2011). The Oxford Foot Model (OFM), which divides the foot into several rigid segments, has shown robust reliability on inter-segmental angles through the gait cycle (Milner and Brindle, 2016; Balsdon and Dombroski, 2018). It has been extensively applied to explore the biomechanical properties of the foot during different movement tasks, such as walking (Sun et al., 2018), running (Xiang et al., 2020a), and jumping (Xiang et al., 2020b). Nevertheless, information concerning the influence of prolonged running activities on foot inter-segment kinematics is still limited.

Kinetic gait parameters, such as ground reaction forces (GRFs) and the corresponding impact loading rate exhibited strong correlations with running distance (Derrick et al., 2002; Morin et al., 2011; Degache et al., 2013). Decreased vertical GRFs and lower vertical average loading rate (VALR) were detected after long-distance running, which was speculated as an indicator that the runner tends to shift the running style toward a smoother pattern to alleviate the increased mechanical stresses and avoid RRI (Morin et al., 2011). Since the OFM is a kinematics-only model, a combination of kinetics with this multi-segment foot model shall yield more insight into foot function during long-distance running. Therefore, the purpose of this study was to investigate the differences in inter-segment foot kinematics and GRFs before (baseline test (0 km)), during (interval test after 5 km), and after (final test after 10 km) long-distance running. It was hypothesized that: 1) the inter-segment foot kinematics would change after 5 and 10 km of running, and that 2) the vertical GRFs and VALR would decrease as running distance increased and some differences may also be detected on horizontal GRFs after 5 and 10 km of running.

## 2 Materials and Methods

### 2.1 Participants

A total of 10 marathon-experienced recreational runners (demographic information: age, 25.63 ± 2.88 years; height, 171.88 ± 3.64 cm; weight, 64.73 ± 5.68 kg; BMI, 21.99 ± 2.75 kg/m^2^; training status: running experiences, 4.25 ± 1.81 years; running frequency, 3.13 ± 0.64 times/week; running distance: 7.88 ± 3.76 km per time) were recruited to participate in this study. All of them have regularly joined in the half or full marathon race within 3 years and they were free from any lower limb and foot injuries at least 6 months before this experiment. All runners prefer a rear-foot strike at the beginning of long-distance running and the dominant leg was confirmed to be the right one based on the kicking test. Informed written consent was obtained and this study was approved by the Ethics Committee in Ningbo University (RAGH20201013).

### 2.2 Experimental Protocol and Procedure

**FIGURE 1:**
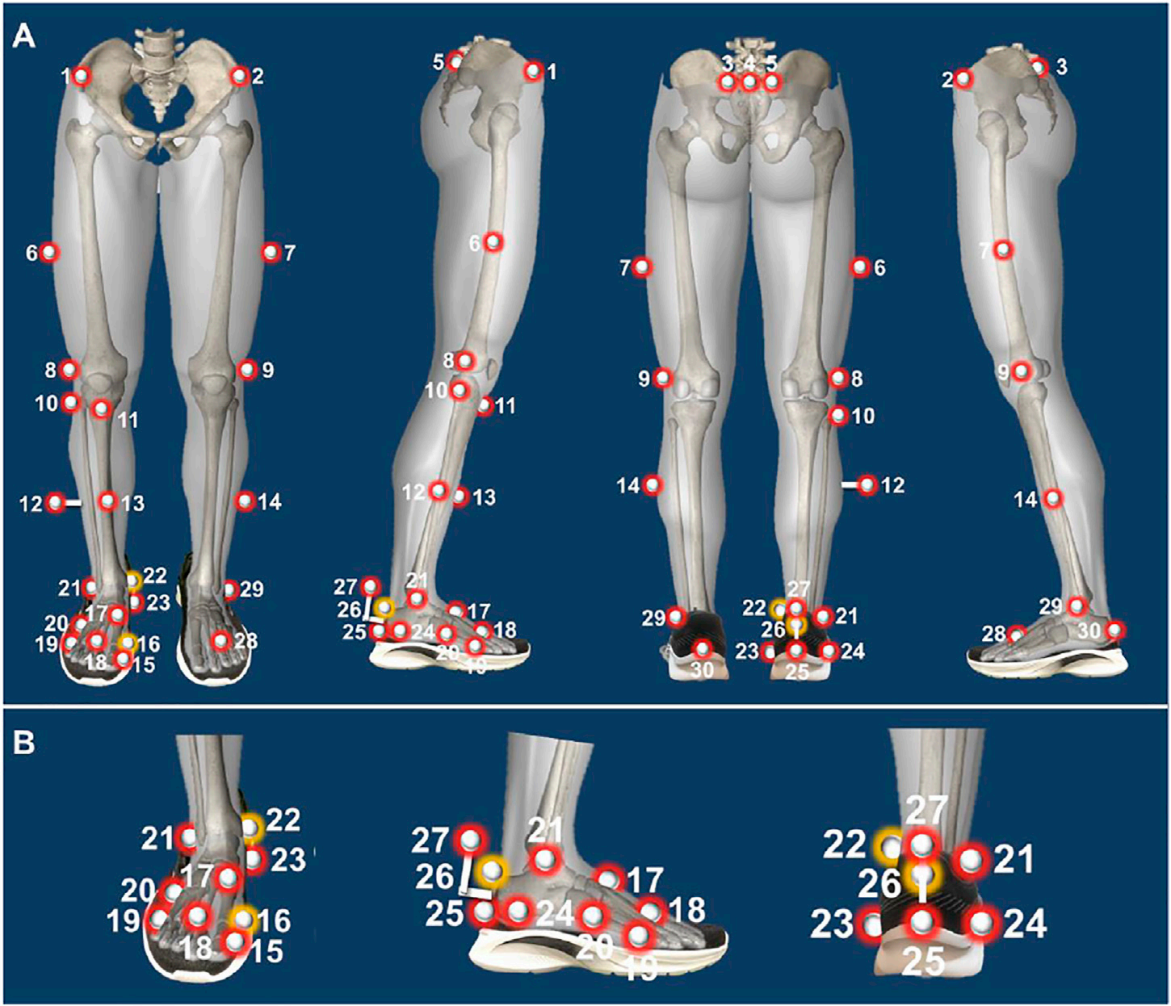
Illustration of Oxford Foot Model marker placement protocol (**(A)**: Lower limb model; **(B)**: Foot multi-segmental model). Note: 1, RASI: right anterior superior iliac spine; 2, LASI: left anterior superior iliac spine; 3, LPSIS: left posterior superior iliac spine; 4, SACR Sacral marker; 5, RPSIS: right posterior superior iliac spine; 6, RTHI: right thigh marker; 7, LTHI: left thigh marker; 8, RKNE: right lateral knee; 9, LKNE: left lateral knee; 10, RHFB: right lateral head of fibula; 11, RTUB: right tibial tuberosity; 12, RTIB: right tibial marker; 13, RSHN: right anterior aspect of the shin; 14, LTIB: left tibial marker; 15, RHLX: right hallux; 16, RD1M: right 1st metatarsal, distal medial; 17, RP1M: right 1st metatarsal, proximal dorsal; 18, RTOE: right toe; 19, RD5M: right 5th metatarsal, distal lateral; 20, RP5M: right 5th metatarsal, proximal lateral; 21, RANK: right ankle; 22, RMMA: right medial malleoli; 23, RSTL: right sustaniculum tali; 24, RLCA: right lateral calcaneus; 25, RHEE: right heel; 26, RPCA: right posterior calcaneus proximal; 27, RCPG: right peg marker; 28, LTOE: left toe; 29, LANK: left ankle; 30, LHEE: left heel; The yellow markers were removed after static calibration.

A Vicon motion capture system (Oxford Metrics Ltd., Oxford, United Kingdom) with 8 infrared cameras was applied to collect the foot inter-segment kinematics during running at 200 Hz. A total of 30 spherical reflective markers with 9 mm in diameter were attached to the corresponding bony landmarks through the hole cut in the shoes based on a previously established protocol (Song et al., 2020), and three markers (LD1M, LMMA, and LCPA) were removed after the static calibration trials (Figure 1). GRFs data and gait cycle were obtained using an AMTI force platform (Advance Mechanical Technology Inc, Watertown, United States) embedded in the middle of a 20-m indoor walkway at a frequency of 1000 Hz. The Vicon Nexus software (Version 1.8.5, Oxford Metrics Ltd., Oxford, United Kingdom) was used to record the kinematics and kinetics data synchronously. The same pants and running shoes were provided and participants were required to wear them while running at least 1 week before the test for adaptation.

The baseline data were first collected during the test day after a 10-min basic warm-up and environment familiarization. Participants were instructed to run on the 20-m indoor track at 3.3 m/s (equal to 12 km/h), with the above motion capture system and force platform used to record data and a timing gate to monitor the running speed. After that, participants were asked to run for 10 km on a treadmill at 12 km/h. This running protocol was chosen because a running distance of 10 km is long enough to initiate changes, and 12 km/h corresponds to the average moderate running speed for recreational runners (Kim et al., 2018). The marker and GRFs data were again measured immediately after 5 km of running. After the interval test, participants kept running for another 5 km and did the final 10-km test.

### 2.3 Data Collection and Processing

Demographic information and training experience were collected and calculated before the test. Gait data were first labelled and run in Vicon Nexus. An experienced technician further checked the traces and removed all the inconsistent trials. Five successful trials for each participant were extracted for further analysis. The dominant foot inter-segment kinematics, including forefoot with respect to hindfoot angles (FFHFA) and hindfoot with respect to tibia angles (HFTBA) in the sagittal, frontal, and transverse planes, as well as hallux with respect to forefoot angle (HXFFA) in the sagittal plane, were measured. Angle values at initial contact (IC) and toe-off (TO), as well as peak values and range of motion (ROM) in the stance phase, were then derived. For GRFs parameters, the 1st and 2nd vertical GRFs, peak propulsive, and breaking GRFs in the stance phase, together with the 1st and 2nd VALR, were extracted. The peak propulsive and breaking GRFs refer to the peak positive and negative GRF in *X*-axis (anteroposterior direction), and VALR were calculated by dividing the corresponding vertical GRF with the time from IC. All kinetic parameters were normalized to body weight for further analysis.

### 2.4 Statistical Analysis

For peak variables analysis, one-way repeated-measures analysis of variance (ANOVA) through SPSS 17.0 software (SPSS, Chicago, IL, United States) was taken to examine the significance of foot inter-segment kinematics and GRFs at critical points between the baseline condition, 5 km, and 10 km of running. The Shapiro-Wilk test was first performed to assess data normality and data were presented as mean ± SD (standard deviation). The mean differences (confidence intervals (95%CI)) among groups were also calculated. Moreover, ANOVA of one-dimensional statistical parametric mapping (SPM1d) was also conducted to further observe foot kinematics changes over the stance phase by using MATLAB 2019b software (The MathWorks, Natick, MA, United States). The statistical significance level was set at *p* < 0.05.

## 3 Results

### 3.1 Foot Inter-segment Kinematics

**FIGURE 2:**
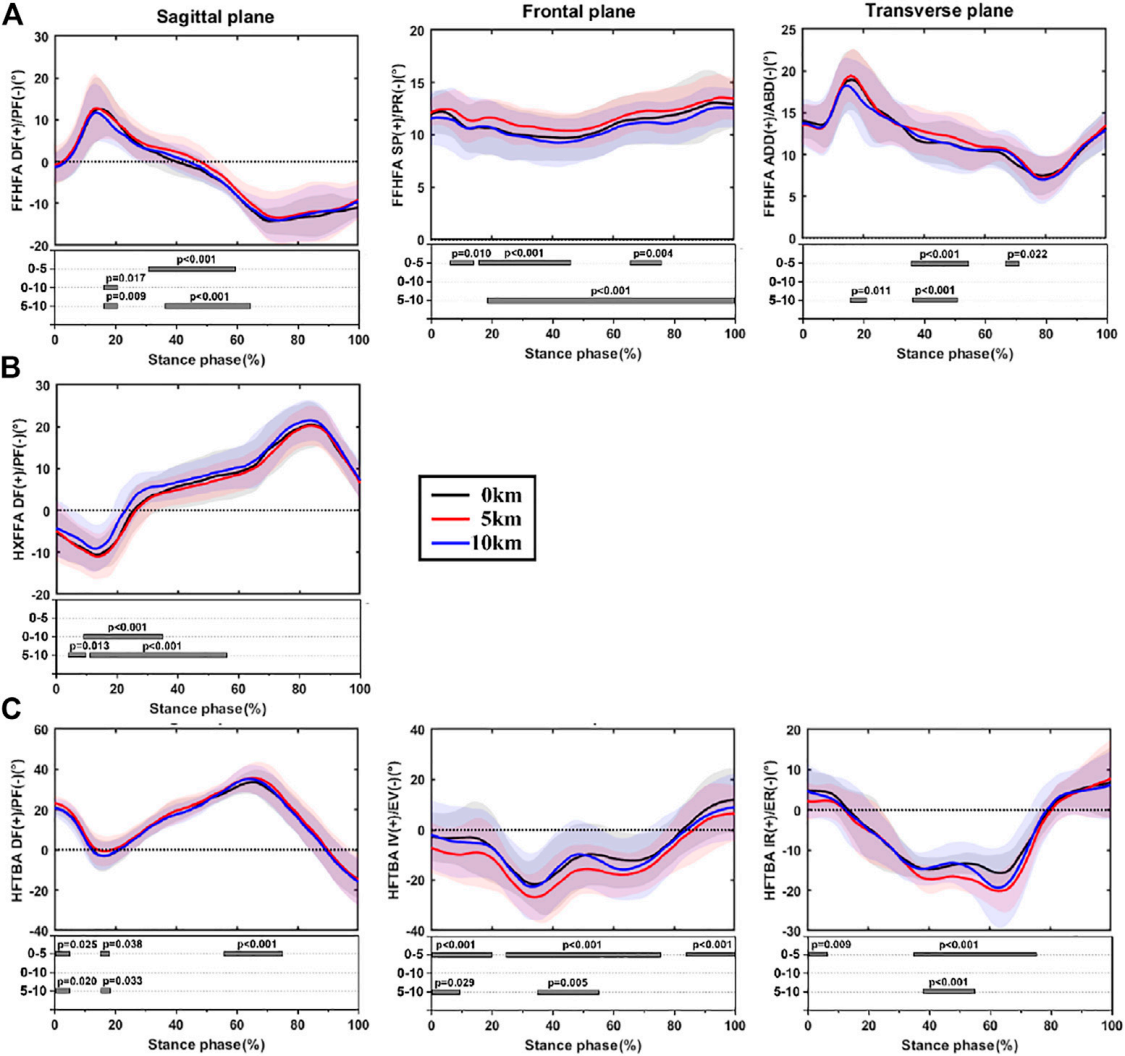
The time-series data and SPM1d analysis of foot inter-segment kinematics during stance phase at baseline condition and immediately after 5 and 10 km of running, **(A)**: forefoot with respect to hindfoot motion; **(B)**: hallux with respect to forefoot motion; **(C)**: hindfoot with respect to tibia motion. Note: FFHFA, forefoot with respect to hindfoot angles; HXFFA, hallux with respect to forefoot angle; HFTBA, hindfoot with respect to tibia angles; DF, dorsiflexion; PF, plantarflexion; SP, supination; PR, pronation; ADD, adduction; ABD, abduction; IV, inversion; EV, eversion; IR, internal rotation; ER, external rotation.

The time-series data of foot inter-segment kinematics and the corresponding SPM1d analysis during the stance phase among three conditions are shown in Figure 2. Table 1, Table 2, Table 3 exhibits test statistics for all kinematic parameters at critical points.

**TABLE 1:** Forefoot with respect to hindfoot motion kinematics at baseline and immediately after 5 and 10 km of running.

	Running distance			*p*-values, mean difference (95%CI)		
	Baseline	5 km	10 km	Baseline/5 km	Baseline/10 km	5km/10 km
	Mean (SD)	Mean (SD)	Mean ± SD			
**FFHFA (°)**						
**X**						
IC	−1.30 ± 3.88	−0.77 ± 4.63	−1.29 ± 3.47	0.470, −0.53 (−1.43 to 0.38)	1.000, −0.01 (−0.80 to 0.78)	0.458, 0.52 (−0.37–1.40)
TO	−10.55 ± 2.53	−8.11 ± 4.83	−8.54 ± 3.94	**0.007**, −2.45 (−4.33 to −0.57)	**0.013**, −2.01 (−3.69 to −0.34)	1.000, 0.44 (−0.74–1.61)
DF (max)	13.98 ± 6.44	14.14 ± 6.79	13.42 ± 5.45	1.000, −0.16 (−1.24 to 0.92)	0.561, 0.56 (−0.48–1.60)	0.252, 0.72 (−0.29–1.74)
PF(MAX)	−16.35 ± 2.65	−14.96 ± 6.21	−15.46 ± 5.10	0.125, −1.39 (−3.03 to 0.26)	0.278, −0.89 (−2.17 to 0.40)	0.369, 0.50 (−0.29–1.29)
ROM(DF/PF)	30.33 ± 6.30	29.10 ± 7.45	28.88 ± 6.03	0.304, 1.23 (−0.59–3.04)	0.071.1.45 (−0.09–2.98)	1.000, 0.22 (−1.04–1.48)
**Y**						
IC	11.95 ± 1.84	12.07 ± 1.28	11.52 ± 2.55	1.000, −0.12 (−0.75 to 0.52)	0.172, 0.43 (-0.12–0.99)	0.368, 0.55 (−0.32–1.42)
TO	12.92 ± 2.26	13.38 ± 2.16	12.56 ± 1.74	0.104, −0.46 (−0.98 to 0.07)	0.601, 0.36 (-0.33–1.06)	**0.001**, 0.82 (0.29–1.35)
SP(max)	13.93 ± 2.93	14.41 ± 2.21	13.46 ± 2.17	0.060, −0.48 (−0.98 to 0.01)	0.141, 0.47 (-0.10–1.03)	**< 0.001**, 0.95 (0.50–1.40)
ROM(SP/PR)	4.61 ± 1.12	4.64 ± 1.25	4.66 ± 0.82	1.000, −0.04 (−0.46 to 0.39)	1.000, −0.05 (−0.60 to 0.49)	1.000, −0.02 (−0.61 to 0.57)
**Z**						
IC	14.01 ± 2.07	13.52 ± 2.37	13.70 ± 2.85	0.143, 0.50 (−0.11–1.10)	0.501, 0.31 (−0.24–0.87)	1.000, −0.18 (−0.73 to 0.37)
TO	14.04 ± 2.26	14.40 ± 1.68	13.89 ± 2.42	0.177, −0.37 (−0.83 to 0.10)	1.000, 0.15 (−0.42–0.71)	0.127, 0.51 (−0.10–1.12)
ADD (max)	20.00 ± 3.60	20.66 ± 3.20	19.57 ± 3.54	**0.049**, −0.66 (−1.32 to -0.01)	0.775, 0.43 (−0.50–1.35)	**0.002**, 1.09 (0.34–1.83)
ROM(ADD/ABD)	13.02 ± 2.16	14.04 ± 1.81	12.96 ± 1.92	**0.001**, −1.02 (−1.67 to −0.38)	1.000, 0.06 (−0.96–1.08)	**0.007**, 1.09 (0.24–1.93)

Note: FFHFA, forefoot with respect to hindfoot angles; SD, standard deviation; CI: confidence interval; IC, initial contact; TO, toe-off; DF, dorsiflexion; PF, plantarflexion; SP, supination; PR, pronation; ADD, adduction; ABD, abduction; ROM, range of motion.

**TABLE 2:** Hallux with respect to forefoot motion kinematics at baseline and immediately after 5 and 10 km of running.

	Running distance			*p*-values, mean difference (95%CI)		
	Baseline	5 km	10 km	Baseline/5 km	Baseline/10 km	5km/10 km
	Mean (SD)	Mean (SD)	Mean ± SD			
**HXFFA (°)**						
**X**						
IC	−5.41 ± 5.37	−4.99 ± 7.34	−4.22 ± 6.78	1.000, −0.41 (−1.86 to 1.04)	0.055, −1.18 (−2.38 to 0.02)	0.420, −0.77 (−2.04 to 0.50)
TO	3.37 ± 4.81	2.28 ± 4.69	3.77 ± 5.12	0.452, 1.10 (−0.77–2.96)	1.000, −0.40 (−2.00 to 1.21)	0.180, −1.49 (−3.42 to 0.43)
DF (max)	21.39 ± 5.52	21.02 ± 4.37	22.28 ± 4.74	1.000, 0.37 (−1.13–1.87)	0.214, −0.89 (−2.09 to 0.31)	0.102, −1.26 (−2.69 to 0.17)
PF(max)	−10.87 ± 4.03	−11.40 ± 5.42	−9.79 ± 5.40	0.739, 0.54 (−0.60–1.67)	0.074, −1.08 (−2.24 to 0.08)	**0.006**, −1.62 (−2.85 to −0.38)
ROM(DF/PF)	32.25 ± 5.02	32.42 ± 3.84	32.06 ± 4.20	1.000, −0.17 (−1.24 to 0.91)	1.000, 0.19 (−1.10–1.48)	1.000, 0.36 (−0.78–1.50)

Note: HXFFA, hallux with respect to forefoot angle; SD, standard deviation; CI: confidence interval; IC, initial contact; TO, toe-off; DF, dorsiflexion; PF, plantarflexion; ROM, range of motion.

**TABLE 3:** Hindfoot with respect to tibia kinematics at baseline and immediately after 5 and 10 km of running.

	Running distance			*p*-values, mean difference (95%CI)		
	Baseline	5 km	10 km	Baseline/5 km	Baseline/10 km	5km/10 km
	Mean (SD)	Mean (SD)	Mean ± SD			
**HFTBA (°)**						
**X**						
IC	20.96 ± 3.13	23.19 ± 3.71	20.78 ± 4.48	**0.002**, −2.23 (−3.72 to −0.73)	1.000, 0.19 (−1.33–1.70)	**0.002**, 2.41 (0.81–4.02)
TO	−16.03 ± 9.05	−16.66 ± 13.00	−18.08 ± 12.18	1.000, 0.63 (−2.30–3.57)	0.205, 2.05 (−0.68–4.77)	0.077, 1.42 (−0.11–2.94)
DF (max)	34.82 ± 5.59	37.62 ± 5.96	36.98 ± 5.84	**< 0.001**, −2.79 (−4.04 to −1.54)	**0.005**, −2.15 (−3.76 to −0.55)	0.976, 0.64 (−0.96–2.24)
PF(max)	−16.29 ± 8.80	−16.78 ± 12.78	−18.23 ± 11.89	1.000, 0.50 (−2.30–3.29)	0.194, 1.94 (−0.61–4.49)	**0.047**, 1.45 (0.01–2.88)
ROM(DF/PF)	51.11 ± 8.38	54.40 ± 11.47	55.20 ± 10.12	**0.031**, −3.29 (−6.35 to −0.24)	**0.001**, −4.10 (−6.64 to −1.56)	0.997, −0.81 (−2.85 to 1.23)
**Y**						
IC	−2.72 ± 9.85	−7.14 ± 10.33	−1.92 ± 13.82	**< 0.001**, 4.42 (1.93–6.91)	1.000, −0.81 (−5.04 to 3.43)	**0.008**, −5.23 (−9.30 to −1.15)
TO	11.86 ± 12.84	6.27 ± 10.31	8.96 ± 13.80	**< 0.001**, 5.59 (3.38–7.80)	0.583, 2.90 (−2.56–8.35)	0.325, −2.70 (−6.79 to 1.39)
IV(max)	12.83 ± 13.06	8.14 ± 11.59	10.90 ± 12.35	**< 0.001**, 4.70 (2.81–6.58)	0.953, 1.94 (−2.82–6.69)	0.267, −2.76 (−6.70 to 1.18)
EV (max)	−24.54 ± 11.84	−28.92 ± 11.26	−24.75 ± 13.37	**< 0.001**, 4.38 (2.02–6.74)	1.000, 0.20 (−2.85–3.25)	**0.019**, −4.18 (−7.81 to −0.54)
ROM(IV/EV)	37.38 ± 11.47	37.06 ± 10.82	35.64 ± 8.49	1.000, 0.31 (−2.86–3.49)	0.543, 1.73 (−1.43–4.89)	0.161, 1.42 (−0.36–3.19)
**Z**						
IC	4.78 ± 4.87	2.18 ± 4.71	4.70 ± 6.77	**0.001**, 2.60 (0.96–4.24)	1.000, 0.08 (−1.90–2.05)	**0.006**, −2.52 (−4.44 to −0.60)
TO	7.38 ± 9.36	8.81 ± 10.23	6.69 ± 8.82	0.053, −1.43 (−2.86 to 0.01)	0.578, 0.69 (−0.60–1.98)	**0.014**, 2.12 (0.35–3.88)
IR (max)	10.62 ± 5.92	11.40 ± 7.18	9.08 ± 7.46	0.713, −0.78 (−2.39 to 0.84)	0.060, 1.54 (−0.05–3.13)	**0.017**, 2.32 (0.34–4.30)
ER (max)	−18.55 ± 3.32	−22.30 ± 4.70	−22.24 ± 7.36	**< 0.001**, 3.75 (2.34–5.17)	**< 0.001**, 3.69 (1.55–5.83)	1.000, −0.06 (−2.05 to 1.93)
ROM(IR/ER)	29.17 ± 8.16	33.70 ± 8.87	31.32 ± 10.98	**< 0.001**, −4.53 (−6.47 to −2.59)	0.095,−2.15 (−4.55 to 0.26)	0.128, 2.38 (−0.46–5.22)

Note: HFTBA, hindfoot with respect to tibia angles; SD, standard deviation; CI: confidence interval; IC, initial contact; TO, toe-off; DF, dorsiflexion; PF, plantarflexion; IV, inversion; EV, eversion; IR, internal rotation; ER, external rotation; ROM, range of motion.

#### 3.1.1 Forefoot with Respect to Hindfoot Motion

Through the SPM1d analysis, it was found that FFHFA was significantly different after 5 km of running compared to baseline and 10 km (Figure 2A). The larger dorsiflexion and adduction angles during the mid-stance phase and the larger supination throughout the stance phase were found at 5 km. In addition, 10 km of running resulted in relatively smaller dorsiflexion at the early stance phase compared to baseline and 5 km. In the meantime, significantly less plantarflexion was also found after 5 and 10 km of running by comparing the angles at TO with baseline. The critical point differences among conditions can be found in Table 1.

#### 3.1.2 Hallux with Respect to Forefoot Motion

As for HXFFA in the sagittal plane, smaller plantarflexion angles were presented during the early to midstance phase (Figure 2B) at 10 km of running, and the peak value of plantarflexion also decreased after 10 km of running when compared to baseline and 5 km of running (Table 2).

#### 3.1.3 Hindfoot with Respect to Tibia Motion

Similar trends were also found for hindfoot with respect to tibia motion by examining the full-time series of angles (Figure 2C), especially in the frontal and transverse planes, 5 km of running led to larger eversion and external rotation angles during the mid-stance phase. A consistent result was also found after 5 km of running when comparing the peak angles, angles at IC and TO instant, and ROM. Additionally, some significant differences were presented at 10 km compared to baseline and 5 km. The critical point differences among conditions are shown in Table 3.

### 3.2 Ground Reaction Forces

The group average and statistics of GRFs during the stance phase are presented in Table 4. No significant differences were found among conditions except peak propulsive, with its value decreasing significantly after 10 km compared to 5 km of running.

**TABLE 4:** Ground reaction forces at baseline and immediately after 5 and 10 km of running.

	Running distance			*p*-values, mean difference (95%CI)		
	Baseline	5 km	10 km	Baseline/5 km	Baseline/10 km	5km/10 km
GRFs	Mean (SD)	Mean (SD)	Mean ± SD			
First peak vertical (BW)	1.95 ± 0.28	1.99 ± 0.29	2.03 ± 0.26	1.000, −0.04 (−0.15 to 0.08)	0.268, −0.08 (−0.20 to 0.04)	0.837, −0.05 (−0.16 to 0.06)
Second peak vertical (BW)	2.67 ± 0.18	2.66 ± 0.21	2.60 ± 0.27	1.000, 0.01 (−0.05–0.07)	0.229, 0.07 (−0.02–0.15)	0.134, 0.06 (−0.01–0.12)
First VALR (BW/s)	45.64 ± 15.69	48.11 ± 14.04	47.58 ± 11.04	0.684, −2.47 (−7.48 to 2.54)	1.000, −1.94 (−8.04 to 4.16)	1.000, 0.53 (−4.64–5.69)
Second VALR (BW/s)	27.14 ± 4.59	28.14 ± 3.29	27.85 ± 4.53	0.599, −1.00 (−2.90 to 0.91)	1.000, −0.71 (−2.58 to 1.17)	1.000, 0.29 (−1.37–1.96)
Peak braking (BW)	−0.17 ± 0.05	−0.18 ± 0.07	−0.18 ± 0.08	1.000, 0.01 (−0.02–0.04)	1.000, 0.01 (−0.03–0.04)	1.000, −0.01 (−0.03 to 0.03)
Peak propulsive (BW)	0.14 ± 0.07	0.14 ± 0.08	0.12 ± 0.07	0.741, −0.01 (−0.02 to 0.01)	0.443, 0.01 (−0.01–0.03)	**0.005**, 0.02 (0.01–0.03)

**Note**: SD, standard deviation; CI: confidence interval; GRFs, ground reaction forces; VALR, vertical average loading rate; BW, body weight.

## 4 Discussion

This study set out to measure the inter-segment foot kinematics and GRF differences after a continuous 5 and 10 km of running compared to the baseline. Consistent with our first hypothesis, significant kinematic deviations among conditions were found. However, the second hypothesis was partially supported since only the difference in propulsive force was detected.

### 4.1 Forefoot Motion

By exploring the forefoot motion with respect to rearfoot, it is possible to gain further insight into the plantar dynamic changes during long-distance running. In the sagittal plane, the plantarflexion angle at TO was found to significantly decrease both after 5 and 10 km of running. Although the differences seem relatively small at first glance (2.45° between baseline and 5 km, 2.01° between baseline and 5 km), several studies have revealed that the intricate foot inter-segment movement could have a significant effect on the plantar fascia function (Ferber and Benson, 2011; Michael E Graham et al., 2011; Chang et al., 2014). For instance, a tiny distance change between the forefoot and rearfoot (< 1 mm) would contribute to a 34.8% change of plantar fascia strain (Michael E Graham et al., 2011). Also, a change of 1° in arch angle can lead to a plantar fascia tension change of 0.4–0.7 times of body weight during the early stance phase (Ferber and Benson, 2011). Thus, we speculated that the decreased forefoot plantarflexion motion during the stance phase may result in the increased stress and strain of plantar aponeurosis and consequently higher injury risks. In addition, smaller forefoot dorsiflexion angles were found during the early (15–20%) stance phase at 10 km of running compared to baseline and 5 km of running, which indicates that runners may tend to a relative midfoot strike pattern after long-distance running, probably because of compensating for local muscle fatigue (Kim et al., 2018).

Concerning forefoot motion in the frontal plane, decreased supination angles were observed throughout the stance phase after 10 km of running compared to 5 km, which may also correlate with the changes of foot posture after long-distance running (Mei et al., 2019). Specifically, it has been proved that long-distance running would result in a more pronated foot posture and a redistributed forefoot plantar load with increased pressure under the 2nd and 3rd metatarsal while decreasing under 4th and 5th metatarsals (Bisiaux and Moretto, 2008; Mei et al., 2019). Moreover, the significantly reduced peak forefoot adduction angle and ROM, as well as relatively smaller forefoot adduction during early (15–21%) and mid-stance (37–51%) phase at 10 km of runnimg, could be another possible explanation for the above findings since forefoot adduction is a part of foot supination during the propulsion phase (Levinger et al., 2010). Similar kinematic changes in frontal and transverse planes were also found in individuals with flat arch during walking (Hunt and Smith, 2004). It has been demonstrated that the pronated foot posture after long-distance running could further lead to a reduced arch height (Fukano et al., 2018), which could let the foot move with a relatively “flat arch” pattern and may induce plantar pain during running.

### 4.2 Hallux Motion

Compared to previous data, a relatively different result of hallux motion was observed in this study. Generally, rearfoot-strike runners would present great hallux dorsiflexion during the early and last stance phase of running because of the rollover mechanism (Samson et al., 2014). However, our findings showed that, although there was no significant difference among conditions, the hallux exhibited considerably smaller dorsiflexion angle both at IC and TO. A possible explanation for this difference may be the narrow forefoot part of footwear due to modern aesthetic needs. Previous research has demonstrated that the insufficient forefoot space (width and height) may limit the ambulatory function of toes, affect its kinematic performance during locomotion, and potentially lead to foot injuries (such as bruised toenails) because of repetitive friction (Runners Connect, 2013; Wallden, 2016; Xiang et al., 2018). Two studies measuring the foot inter-segment kinematics while walking or running barefoot presented normal hallux dorsiflexion during the stance phase (Sun et al., 2018; Xiang et al., 2020a). Therefore, more comparisons concerning the effects of forefoot space of footwear on hallux biomechanics are warranted. In addition, the strike pattern may also contribute to this difference. Although all participants in this study preferred rearfoot strike at the beginning of the long-distance running test, it was demonstrated above that runners may shift to a relative midfoot landing strategy as indicated by the smaller forefoot dorsiflexion angles from early to mid-stance phases. Compared to baseline and 5 km of running, it was also found that the plantarflexion angles decreased during the early stance phase at 10 km of running. Together with the smaller peak propulsive force found after 10 km of running, it was speculated that the toes’ dynamic control function, such as gripping, may gradually reduce after long-distance running, which was also consistent with previous findings (Kim et al., 2018; Mei et al., 2018). Nevertheless, more studies concerning the hallux biomechanics during long-distance running are recommended.

### 4.3 Rearfoot Motion

Most of the distinct effects concerning foot inter-segment kinematics were observed in rearfoot motion. As for the sagittal plane, higher dorsiflexion angles at IC and during the early (0–5%) and mid-late (56–75%) stance phase were presented after 5 km of running, and a higher peak dorsiflexion angle and ROM were presented after both 5 and 10 km of running. Although stride length was not measured in this study, it was previously demonstrated that the increase in rearfoot dorsiflexion resulted in a greater stride length (Sun et al., 2018). Similarly, the decreased dorsiflexion angle at IC and increased peak plantarflexion angle, and larger plantarflexion during the early (15–18%) stance phase may indicate the stride length was reduced again after 10 km of running when compared to 5 km. As muscular fatigue may happen during long-distance running, the lower limb is likely to shift its gait to a shorter but quicker step frequency (Millet et al., 2009; Kim et al., 2018), which could be a possible explanation for the stride length change at 10 km of running.

In the frontal plane, higher eversion angles throughout the stance phase were found after 5 km of running compared to the other two conditions. Previous research provided evidence that increased rearfoot eversion motion would be an injury indicator, which may potentially increase the risk of plantar fasciitis because of excessive use (Ryan B Graham et al., 2011; Chang et al., 2014). Moreover, excessive rearfoot eversion would further lead to excessive midfoot dorsiflexion, and this abnormal kinematic coupling foot motion has been proved a risk factor for lower limb RRI (Takabayashi et al., 2018). In addition, greater internal to external rotation, especially during the mid-late stance (34–75%) phase and consequently increased ROM in the transverse plane, were also observed at 5 km of running. An increase in the rearfoot rotation was demonstrated to be associated with an increase in tibial rotation and then would further affect the proximal joints’ function (such as the knee joint) because of the coupling motion (Lundberg et al., 1989; Williams et al., 2001; Levinger et al., 2010). This could also serve as an underlying mechanism for knee injuries after long-distance running, such as iliotibial band syndrome (Aderem and Louw, 2015).

### 4.4 Ground Reaction Forces

A comparison with previous data also revealed some different results in GRFs in our study. Kim et al. (2018) conducted a systematic review to investigate the effect of long-distance running on lower-limb biomechanical parameters in healthy runners. They summarized that lower vertical GRFs and loading rate would be presented after long-distance running due to the increased mechanical stress with decreased musculoskeletal capacities. However, no significant differences among conditions were found in this study except a lower peak propulsive force at 10 km of running compared to 5 km. The participants’ heterogeneity between studies would be the primary explanation for these differences since the fatigue-related changes were speculated to initiate at 10 km of running according to our findings in kinematics. Investigating longer-distance running (such as 20 km) based on participants involved in this study will be performed for further verification. Moreover, the inconsistent GRF results of this study may also be due to the equipment accuracy and future studies using more precise devices (e.g., insole-type pressure mat) could add insight (Lung et al., 2008; Jung et al., 2014).

### 4.5 Limitations

Some limitations of this study should be considered. Firstly, the main purpose of the current study was to investigate the effect of long-distance running on foot adaptions, thus many other parameters, such as running surface, running shoes, and gender were not addressed. Secondly, the plantar pressure differences and the repeated stress loading on soft tissue stiffness before and after long-distance running were not measured, which may add more conclusive explanations for the findings of this study (Pu et al., 2018; Duan et al., 2021). Moreover, the muscular fatigue after 10 km of running was only speculated based on previous research results and further investigation about muscle activity before and after running is warranted for verification. In addition, distinct individuals may show different foot kinematic responses during running and a cluster analysis may help find the particular baseline patterns among them (Watari et al., 2021). Lastly, we only investigated the foot kinematic differences during the stance phase as it is the only time for foot–ground interaction. However, the swing phase kinematics shall present more preparatory foot adaptations during long-distance running (Dixon et al., 2011). Further large sample size studies concerning these aspects would reveal more profound knowledge.

## 5 Conclusion

In summary, this study revealed that the foot inter-segment kinematics and GRFs were significantly influenced by long-distance running. Excessive foot motion after 5 km of running may potentially increase the risks of RRI, while 10 km of running changed foot posture, decreased propulsive force, and may also result in high RRI risks because of muscle fatigue. In addition, the forefoot space of footwear may affect foot biomechanics in response to long-distance running, specifically in the hallux region. Findings from the current study give further insight into how inter-segments of foot interact and how GRFs vary during a long-distance running event, adding references for future studies aiming at foot RRI evaluation and prediction.

## Data Availability

The original contributions presented in the study are included in the article/Supplementary Material, further inquiries can be directed to the corresponding authors.
